# Pauci-Immune Crescentic Glomerulonephritis in Connective Tissue Disease

**DOI:** 10.1155/2016/9070487

**Published:** 2016-07-18

**Authors:** Supraja Yeturi, Mary Cronin, Adam Robin, Campbell Lorna, Ann K. Rosenthal

**Affiliations:** Division of Rheumatology, Department of Medicine, Medical College of Wisconsin, Milwaukee, WI 53226, USA

## Abstract

Pauci-immune crescentic glomerulonephritis is commonly seen in ANCA-associated vasculitis but it is rarely seen during the course of other connective tissue diseases like lupus or Sjogren's syndrome or MCTD. We report 3 cases of pauci-immune crescentic glomerulonephritis in patients with connective tissue disease other than vasculitis. We reviewed literature and made summary of previously reported cases of this rare entity. Clinical and laboratory features of these patients varied widely, but most of patients have met criteria for lupus. In this small population of patients there is no correlation with ANCAs. Most of the patients were treated with aggressive immunosuppression and did well if they were treated early in the course of their disease. One of our patients required renal transplant, but she presented late in the course of her disease, as evidenced by chronicity on her renal biopsy. Whether these patients are overlap of vasculitis and other connective tissue diseases or to be considered as a separate entity is yet to be described. Clinicians must be aware of these presentations because initial presentation can be severe.

## 1. Introduction

Pauci-immune crescentic glomerulonephritis (CrGN) is one of the most common causes of rapidly progressive glomerulonephritis. It is seen as part of small vessel vasculitides, including granulomatosis with polyangiitis, microscopic polyangiitis, and eosinophilic granulomatosis with polyangiitis, or as renal limited vasculitis. Association of pauci-immune CrGN with connective tissue diseases (CTDs) other than vasculitis is rare and only a few case reports have been published. The criteria for the pauci-immune entity of CrGN have been defined as “low intensity of glomerular immunoglobulin staining by direct immunofluorescence (IF) assay in renal sections in the negative to 1+ range on a scale of 0 to 4+” [1]. Glomerulonephritis is commonly associated with lupus but the occurrence of pauci-immune CrGN is rare in lupus and connective tissue diseases other than vasculitis [2–4].

We present 3 cases of pauci-immune CrGN associated with CTDs other than systemic vasculitis. A pathologist reviewed the tissue sections, electron microscopy, and IF of these patients.

## 2. Case  1

A 31-year-old Caucasian woman was diagnosed with lupus in 2011. She initially presented with arthritis, serositis (pleuritis), autoimmune hemolytic anemia, idiopathic thrombocytopenia (ITP), positive antinuclear antibody (ANA), Smith and RNP antibodies, and low complement levels. She also has a history of autoimmune hepatitis and diabetes mellitus type 1. She was initially treated with intravenous immunoglobulin (IVIG), rituximab, and splenectomy. Her disease was relatively stable on hydroxychloroquine 200 mg BID and mycophenolate mofetil (MMF) 500 mg twice a day. In June 2013, she presented with complaints of dyspnea, lower extremity swelling, fevers, anorexia, and jaundice.

Initial workup revealed worsening of chronic anemia with hemoglobin of 5.2 g/dL (her baseline, 8–10 g/dL, and normal, 11.2–15.7 g/dL), hyperbilirubinemia with total bilirubin of 9.2 mg/dL (normal, 0.2–1.2 mg/dL), direct bilirubin of 6.4 mg/dL (normal, 0.0–0.3 mg/dL), alkaline phosphatase of 250 *μ*/L (normal, 35–104 *μ*/L), normal ALT and AST, and a right-sided pleural effusion on chest X-ray. She was treated for a flare of autoimmune hemolytic anemia with IVIG, blood transfusions, intravenous pulse doses of methylprednisolone, empiric treatment with broad spectrum antibiotics for pleural effusion, and continuation of the home dose of hydroxychloroquine 200 mg daily and MMF 500 mg twice a day. Antibiotics were stopped after pleural fluid and blood cultures were found to be negative. Liver biopsy showed iron overload in hepatocytes and no significant inflammation or fibrosis.

Her creatinine increased from a baseline of 0.8 to 1.8 mg/dL and peaked at 2.81 mg/dL (normal, 0.5–1.10 mg/dL) on the fifth day of hospitalization. Urinalysis showed large protein and blood, and urine microscopy showed many red blood cells (RBCs) with few dysmorphic RBCs and many granular casts, fatty casts, and white blood cell casts but no RBC casts. Urine random protein/creatinine ratio was elevated at 2.49 (normal < 0.15).

**Figure 1:**
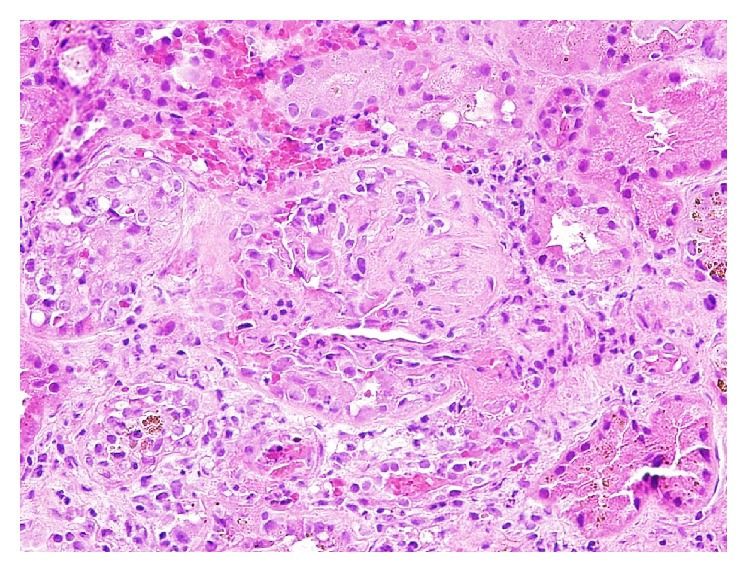
Case  1. Glomeruli with fibrocellular crescent and segmental necrosis. Hematoxylin & Eosin, 200x magnification.

Renal biopsy (Figure 1) showed glomeruli with cellular and fibrocellular crescents, focal segmental necrosis, and mild interstitial and tubular injury. IF showed only trace C3, IgM, and fibrinogen deposits. Electron microscopy (EM) showed endothelial and podocyte injury with glomerular basement membrane (GBM) remodeling; no electron dense immune deposits were seen. The findings were consistent with pauci-immune crescentic glomerulonephritis with focal acute activity and mild chronic changes.

Additional laboratory testing revealed a positive ANA with a titer of 1 : 640 (homogenous pattern), elevated anti-Smith antibody of >8.0 (normal, 0.0–0.9 AI), RNP antibody of 7.2 (normal, 0.0–0.9 AI), p-ANCA at >1 : 640 (normal, <1 : 20), anti-myeloperoxidase (MPO) antibodies at 86.0 U/mL (normal, 0.0–9.0 U/mL), anticardiolipin IgM at 27 U/mL (normal, 0–12 U/mL), and positive lupus anticoagulant. She had a low C3 level of 56 mg/dL (normal, 90–180 mg/dL) and normal C4 level. Anti-double stranded DNA antibodies (dsDNA), c-ANCA, anti-proteinase 3 (PR-3) antibodies, and beta-2 glycoprotein antibodies were negative.

Based on biopsy results, MMF was switched to cyclophosphamide 175 milligrams daily orally. Creatinine returned to normal and her anemia improved. She was tapered off of cyclophosphamide and switched to azathioprine for maintenance therapy.

## 3. Case  2

A 58-year-old African American woman was diagnosed with mixed connective tissue disease (MCTD) in 2000. She initially presented with Raynaud's phenomenon, myalgia, synovitis, esophageal dysmotility, and positive anti-RNP antibody. She also had interstitial lung disease with a nonspecific interstitial pneumonitis (NSIP) pattern and was treated with cyclophosphamide. She has no sclerodactyly or skin findings suggestive of scleroderma. She was hospitalized at our institution in September 2009 for acute renal failure with a creatinine level of 5.52 mg/dL (normal, 0.5–1.10 mg/dL); her baseline was 1.12 mg/dL. Other than increased nocturia she had no other symptoms. Urinalysis showed 2+ blood, 2+ protein, and RBC casts. Urine random protein/creatinine ratio was elevated at 1.28 (normal < 0.15). Renal biopsy was performed for further evaluation.

**Figure 2:**
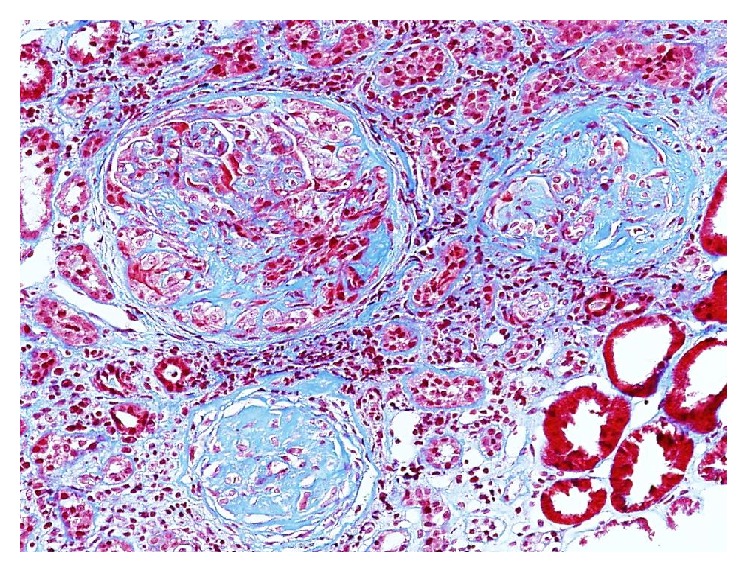
Case  2. One glomerulus with fibrocellular crescent (upper left), two glomeruli with global sclerosis, interstitial inflammation with marked interstitial fibrosis, and tubular atrophy. Masson trichrome, 100x magnification.

Renal biopsy (Figure 2) showed predominantly sclerotic glomeruli with occasional fibrous and fibrocellular crescents and focal segmental necrosis. Marked background tubular atrophy and interstitial fibrosis were also noted. IFE revealed weak, nonspecific reactivity for C3 and fibrinogen. Electron microscopy showed glomerular damage and rare electron dense C3 deposits. The findings were consistent with pauci-immune crescentic glomerulonephritis with focal acute activity and marked chronic changes.

Additional laboratory workup revealed that ANA was high at 64 by EIA (normal, 0–10 U/mL). C3 and C4 were within normal limits. Anti-double stranded DNA antibody, anti-centromere antibody, anti-topoisomerase antibody, anti-JO 1 antibody, anti-ribosomal P protein antibody, anti-SSA/SSB antibody, anti-Smith antibody, anti-MPO antibody, anti-PR-3 antibody, c-ANCA, p-ANCA, and atypical ANCAs were negative. She received a pulse dose of IV methylprednisolone along with five monthly infusions of cyclophosphamide IV and was transitioned to MMF and low dose oral prednisone. Her renal function continued to worsen, and she was started on hemodialysis and underwent renal transplant in 2014. She remains on MMF, tacrolimus, and prednisone; she has had no recent flares of her MCTD. Her transplanted kidney continues to function well, as demonstrated by a stable creatinine of 1.11–1.17 mg/dL.

## 4. Case  3

A 24-year-old African American female with past medical history of asthma and eczema presented to our emergency room with nausea, vomiting, myalgia, and fever in May 2015. Her mother has rheumatoid arthritis. Physical exam was unremarkable. Initial workup revealed creatinine of 4.25 mg/dL (normal, 0.5–1.10 mg/dL) and WBC count of 20 10e3/*μ*L (normal, 4.0–10.0 10e3/*μ*L). Urinalysis showed 2+ blood, 2+ protein, and WBC and granular casts. Urine random protein/creatinine ratio was elevated at 1.90 (normal < 0.15). Ultrasound of the kidneys showed enlarged and edematous kidneys. Further workup revealed elevated ANA of >8 (normal < 0.9 AI), anti-dsDNA antibody at 32 (normal < 5 IU/mL), and anti-chromatin antibody of 7.3 (normal, 0.0–0.9 AI). Anti-MPO, anti-PR-3, anti-Smith, anti-RNP, and anti-SSA/SSB antibodies, C-ANCA, P-ANCA, lupus anticoagulant, and anti-cardiolipin antibody were negative. C3 and C4 were within normal limits. Kidney biopsy was performed for further evaluation.

**Figure 3:**
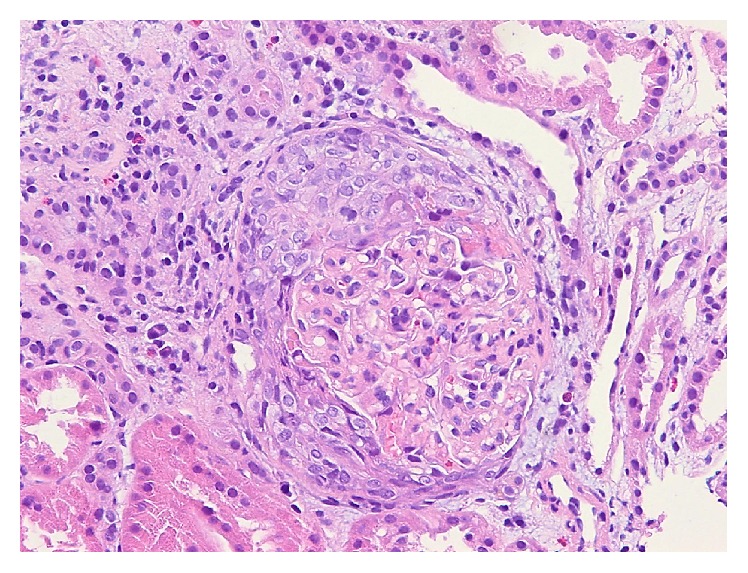
Case  3. Glomeruli with cellular crescent and segmental necrosis. Hematoxylin & Eosin, 200x magnification.

Renal biopsy (Figure 3) showed cellular crescent formation, focal segmental necrosis, and mild interstitial fibrosis. IFE showed sparse positivity for C3 and strong reactivity for fibrinogen within necrotic foci. EM was unremarkable. The findings were consistent with pauci-immune crescentic glomerulonephritis with acute activity and mild chronic changes.

She was treated with pulse dose IV methylprednisolone 1000 mg/day for 3 days followed by oral prednisone at 1 mg/kg/day. She received rituximab 375 mg/m^2^ every week for a total of 4 doses. Her kidney function improved and creatinine returned to normal. She is on rituximab infusions for maintenance therapy and continues to do well.

## 5. Materials and Methods

A literature search was performed in PubMed using the terms “Pauci-immune GN and ANCA in lupus” for all articles published in the last 20 years. We also searched for all the references in the manuscripts retrieved. Criteria for pauci-immune CrGN are defined as “patients with diffuse and focal proliferative glomerulonephritis with necrotizing lesions or crescents, ≤1+ staining on IF and trace deposits on electron microscopy (EM) without thrombotic microangiopathy” [1]. We were able to identify 15 cases of proliferative and necrotizing crescentic glomerulonephritis in connective tissue diseases other than vasculitis. Clinical, laboratory features, and kidney biopsy results of these cases are described in Table 1. The patients presented with a wide variety of clinical features including joint pain, rash, sinus symptoms, and diffuse alveolar hemorrhage. Most of patients had diffuse proliferative glomerulonephritis with crescents and necrotic lesions; kidney biopsy results were not described in one patient. In only 4 cases IF staining was less than +1. All patients had positive ANA and only one patient had ANA titer less than 1 : 80. Anti-dsDNA was positive in 10 patients. ANCAs were negative in 9 patients and positive in 6 patients. In patients with positive ANCA, the pattern is P-ANCA and all of them were positive for anti-MPO. None of patients were positive for anti-PR-3.

## 6. Discussion

Pauci-immune crescentic glomerulonephritis is characterized by focal necrotizing and crescentic glomerulonephritis with little or no glomerular staining for immunoglobulins by immunofluorescence microscopy examination. Pauci-immune crescentic glomerulonephritis is commonly seen in vasculitis and is rarely seen in other connective tissue diseases. Neumann et al. [8] proposed that immunoglobulin deposits can be seen in patients with ANCA-associated CrGN but are not typical for pauci-immune CrGN. CrGN with immune deposits can be seen in connective tissue diseases other than vasculitis but actual pauci-immune CrGN is very rare. We add 3 cases of pauci-immune crescentic glomerulonephritis in patients with connective tissue disease other than vasculitis to this rare entity in the literature.

Patient 1 in our series fulfilled SLICC criteria for lupus [9] but patient 3 did not fulfill lupus criteria. We believe that she is at an early stage in her disease and has yet to develop other clinical manifestations of lupus. Her serology supports the diagnosis of lupus. She does not have any clinical manifestations of vasculitis, her ANCA is negative as are anti-PR-3 and anti-MPO antibodies, and ANCA vasculitis is rare in young African American women [10, 11]. Kidney biopsies on all our patients showed diffuse and focal proliferative crescentic glomerulonephritis with necrotizing lesions and crescents, ≤1+ staining on IF, and trace deposits on electron microscopy (EM) without thrombotic microangiopathy. None of our patients have any evidence of vasculitis.

We found 15 cases in the literature with proliferative and necrotizing crescentic glomerulonephritis in connective tissue diseases other than vasculitis. Most of the patients, except patients reported by Fayaz et al. [2], Akhtar et al. [3], and Li et al. [4], have more than 1+ staining on IF. Only 4 out of 15 patients meet the criteria for pauci-immune crescentic glomerulonephritis. In chronic thrombotic microangiopathy, membranoproliferative GN can be seen without immune deposits [12]. All patients presented by Charney et al. [5] (except patient C) have features of thrombotic microangiopathy on their kidney biopsy. GN in these patients might be a feature of antiphospholipid syndrome, which is treated differently than pauci-immune CrGN. Thrombotic microangiopathy should be in on the differential in patients with CrGN in connective tissue diseases other than vasculitis, and kidney biopsy should look for features of microangiopathy.

Patient 1 in our series has an elevated p-ANCA titer and positive anti-MPO antibody; however, she does not have any common clinical features of systemic small vessel vasculitis. ANCA is detectable in 15–20% of patients with SLE (mainly p-ANCA pattern [13]) and is seen less commonly in other CTDs [14]. Some authors have shown a correlation of ANCA with crescentic lupus nephritis [15, 16], although several others have failed to find a correlation between ANCA and lupus nephritis [17]. Pan et al. have proposed that ANCA might be used as a complementary parameter to differentiate lupus nephritis from SLE without nephritis and also as a marker of disease activity in lupus patients. Yu et al. [18] reported that crescentic glomerulonephritis is seen in 10% of biopsy-proven lupus nephritis and these patients have lower intensity of immunofluorescence <2+ and higher incidence of ANCA positivity. However, they did not comment on whether any of these patients met the criteria for pauci-immune CrGN.

Nasr et al. [15] presented 10 cases of necrotizing and CrGN in patients with lupus, all of whom were seropositive for p-ANCA. All these patients had more than 1+ staining on immunofluorescence, so none of them met the criteria for pauci-immune CrGN. This shows that necrotizing CrGN is commonly seen in lupus patients but pauci-immune CrGN is very rare. Schwartz et al. [19] presented 4 cases of necrotizing glomerulitis, but this study was done before ANCAs were tested; we did not include these cases in our study.

The pathophysiology of pauci-immune CrGN remains unclear. Most authors suggested that cell-mediated immunity plays a more important role in pauci-immune GN than antibody/immune complex mediated glomerulonephritis. Cunningham et al. [20] showed that, in pauci-immune GN, there is development of significant cell-medicated immunity with activated T cells, macrophages, tissue factor, and fibrin at the site of glomerular injury, suggesting that this is most likely a manifestation of T cell-directed cognate immune injury. Activated T cells recruit and activate macrophages, and these sensitized cells can cause severe glomerular injury independent of humoral immune responses. Couser [21] and Li et al. [4] suggested delayed-type hypersensitivity as the underlying mechanism of glomerular damage in ANCA-associated pauci-immune GN.

Patient 1 in our series responded well to corticosteroids and cyclophosphamide but patient 2 progressed to ESRD despite treatment with corticosteroids and cyclophosphamide. We speculate that the reason for her treatment failure is that she presented with advanced renal disease, which was supported by predominant sclerosis and chronic changes on her renal biopsy. Patient 3 was treated with rituximab and corticosteroids; this regimen was considered instead of cyclophosphamide mainly because of her age and fertility concerns. There is no standard treatment protocol for pauci-immune CrGN associated with other CTDs. All patients reported by Akhtar et al. [3], two out of five patients reported by Charney et al. [5], one patient reported by Fayaz et al. [2], one patient reported by Marshall et al. [7], and three out of four patients reported by Hervier et al. [6] responded to corticosteroids and cyclophosphamide. Based on these few patients, no definite conclusions can be made regarding treatment. However, all patients who were presumptively treated for ANCA-associated pauci-immune CrGN had a good response. Renal outcomes were better if they were treated in early stages.

We conclude that CrGN is rarely seen in CTD other than vasculitis, but pauci-immune CrGN without glomerular immune deposits is extremely rare. Cell-medicated immunity with activated T cells is the likely underlying pathogenesis for glomerular injury, and these patients can be treated as ANCA-associated pauci-immune CrGN. Renal outcome is better if patients are treated early in the course of their disease. Improved understanding of risk factors and treatment strategies for CrGN in patients with connective tissue diseases will require larger numbers of patients and further study.

## Figures and Tables

**Table 1:** 

	Clinical features	Kidney biopsy		Antibodies and C3, C4
		Histology	IF and EM findings	
Charney et al. # A [5]	Raynaud's phenomenon, pulmonary fibrosis, esophageal dysmotility, and TTP	DPGN with thrombotic microangiopathy	Trace IgG, IgM, and no C1q, C3, IgA, or fibrinogen. No deposits on EM	Positive ANA with 1 : 640 titer and speckled pattern and lupus anticoagulant. Negative anti-dsDNA and ANCA. Normal C3, C4

Charney et al. # B [5]	ND	DPGN with necrotizing lesions, crescents, and thrombotic microangiopathy	2+ IgM and fibrinogen. Few scattered subepithelial deposits on EM	Positive ANA with 1 : 640 and homogeneous pattern and positive anti-dsDNA. Negative ANCA. Low C3 and C4

Charney et al. # C [5]	Arthritis, leukopenia, coombs-positive hemolytic anemia, and serositis	DPGN with 40% sclerosis and 10% cellular and fibrous crescents	IgM (2+), IgA (1+), C1q (1+), and fibrinogen (1+). Scattered mesangial deposits on EM	Positive ANA with 1 : 640 titer and speckled pattern and anti-dsDNA, SS-A, and SS-B. Negative ANCA. Normal C3, C4

Charney et al. # D [5]	Diagnosed with SLE, features are not described	DPGN and thrombotic microangiopathy	Mild staining for IgG, IgM, and C3	Positive ANA with 1 : 140 titer, homogeneous pattern, and positive dsDNA. Negative ANCA and anticardiolipin. Low C3 and C4

Charney et al. # E [5]	Arthritis	DPGN and thrombotic microangiopathy	Mild IgG and C3. Rare mesangial and intramembranous deposits on EM	Positive ANA with 1 : 320 titer, homogenous pattern, and anti-dsDNA, anticardiolipin IgG. Negative ANCA. Low C3 and normal C4

Hervier et al. # A [6]	Arthritis, hemolytic anemia, thrombocytopenia, sinusitis, cerebral ischemia, and intra-alveolar hemorrhage	Diffuse and global proliferative and crescentic GN	Endomembranous and mesangial IgG, IgM, and C3 deposits	Positive ANA with 1 : 5120 titers, homogenous pattern, anti-dsDNA, and MPO ANCA. Low C3

Hervier et al. # B [6]	Discoid lupus, arthralgia, thrombocytopenia, and epistaxis	Proliferative crescentic GN, without mesangial proliferation or IgG deposits		Positive ANA with 1 : 640 titer, speckled pattern, anti-dsDNA, and MPO ANCA

Hervier et al. # C [6]	Arthritis, serositis, thrombocytopenia, anemia, rhinitis, excavated pulmonary nodules, and leukocytoclastic vasculitis	Proliferative and necrotic GN	IgG and IgA deposits	Positive ANA with 1 : 2560 titer, homogenous pattern, anti-dsDNA, MPO ANCA, lupus anticoagulant, and anticardiolipin. Low C3 and C4

Hervier et al. # D [6]	Discoid lupus, arthritis, pericarditis, thrombocytopenia, and intra-alveolar hemorrhage	ND	ND	Positive ANA with 1 : 160 titer and MPO ANCA. Negative dsDNA. Low C3

Li et al. # A [4]	Rash, hemolytic anemia	DPGN with fibrocellular crescents	No immunofluorescence deposits. Scanty subepithelial electron dense deposits on EM	Positive ANA, 1 : 320 titer, and homogenous pattern. Negative anti-dsDNA, anti-PR-3, and anti-MPO antibody. Low C3 and C4

Akhtar et al. # A [3]	Arthritis, alopecia, and AIHA	Diffuse mesangial cellularity, segmental cellular proliferation with necrosis. No crescents	1+ focal staining for IgG and C3	Positive ANA with 1 : 2560 titer, peripheral pattern, and IgM, IgG anticardiolipin. Negative anti-dsDNA, RF, and ANCA. Low C3 and C4

Akhtar et al. # B [3]	Arthritis, Raynaud's phenomenon, serositis, rash, and oral ulcers	Cellular proliferation of glomeruli with areas of necrosis. No crescents	1+ focal staining in some mesangial areas. Small mesangial deposits	Positive ANA with 1 : 2560 titer, homogenous pattern, anti-dsDNA, and SS-A/SS-B. Negative RF, ANCA. Low C4

Marshall et al. # A [7]	Pancytopenia	Segmental necrotizing GN with cellular crescents	2+ IgG and C3, negative IgM, IgA, and C1. No subendothelial electron dense deposits	Positive ANA, 1 : 160, speckled pattern, anti-dsDNA, anti-histone antibody, and P-ANCA. Negative C-ANCA. Low C3 and normal C4

Marshall et al. # B [7]	Serositis, sinusitis, and pulmonary infiltrates	Diffuse thickening of glomerular basement membranes, segmental necrotizing GN with cellular crescents	3+ IgG, 2+ C3, and trace IgM. No subendothelial electron dense deposits	Positive ANA with 1 : 80 titer, P-ANCA, and MPO antibody. Negative C-ANCA, anti-dsDNA. Normal C3 and C4

Fayaz et al. # A [2]	Arthritis, rash, photosensitivity, and leukopenia	Segmental and focal proliferative fibrinoid necrosis	No IF deposits. No electron dense deposits	Positive ANA with 1 : 640 titer, homogeneous pattern, and anti-dsDNA. Negative ANCA, anti-MPO, and PR-3 antibodies. Low C3, normal C4

TTP: thrombotic thrombocytopenic purpura, IF: immunofluorescence, EM: electron microscopy, AIHA: autoimmune hemolytic anemia, MPO: myeloperoxidase, PR-3: proteinase 3, ND: not described, GN: glomerulonephritis, and DPGN: diffuse proliferative GN.
